# Functional ability in a population: normative survey data and reliability for the ICF based Norwegian Function Assessment Scale

**DOI:** 10.1186/1471-2458-7-278

**Published:** 2007-10-03

**Authors:** Nina Østerås, Søren Brage, Andrew Garratt, Jurate Saltyte Benth, Bård Natvig, Pål Gulbrandsen

**Affiliations:** 1 Section of Occupational Health and Social Insurance Medicine, Institute of General Practice and Community Health, Faculty of Medicine, University of Oslo, Norway; 2 Institute of Health Management and Health Economics, University of Oslo, Norway; 3 Helse Øst Health Services Research Centre, Akershus University Hospital, Norway; 4 Faculty of Medicine, University of Oslo, Norway

## Abstract

**Background:**

The increasing focus on functional ability assessments in relation to sickness absence necessitates the measurement of population functional levels. This study assessed the reliability of the Norwegian Function Assessment Scale (NFAS) and presents normative population data.

**Methods:**

All inhabitants in seven birth cohorts in Ullensaker municipality in 2004 were approached by means of a postal questionnaire. The NFAS was included as part of The Ullensaker Study 2004. The instrument comprises 39 items derived from the activities/participation component in the International Classification for Functioning, Disabilities and Health (ICF). Based on the results of principal component analysis, these items comprise seven domains. Non-parametric tests for independent samples were used to compare subgroups. Internal consistency was assessed by Cronbach's alpha. Two-week test-retest reliability was assessed by total proportions of agreement, weighted kappa, and intraclass correlation coefficient (ICC).

**Results:**

The response rate was 54% (1620 persons) and 75.4% (101 persons) for the retest. Items had low levels of missing data. Test-retest reliability was acceptable with high proportions of absolute agreement; kappa and ICC values ranged from 0.38 to 0.83 and 0.79 to 0.83, respectively. No difficulty on all 39 functional activities was reported by 33.1% of respondents. Females, older persons and persons with lower levels of education reported more functional problems than their respective counterparts (p < 0.05). The age gradient was most evident for three of the physical domains. For females aged 24–56 and males aged 44–76, a clear education gradient was present for three of the physical domains and one mental domain after adjusting for age and gender.

**Conclusion:**

This study presents population based normative data on functional ability, as measured by the NFAS. These data will serve as basis for the development of national population norms and are necessary for score interpretation. Data quality and test-retest reliability of the NFAS were acceptable.

## Background

Longitudinal trends in sickness absence and disability pensions rates in several European countries, including Norway, show that increasing proportions of the population have levels of work ability that are too low to meet work demands [1]. To meet this challenge, European social security schemes increasingly emphasize the individual's resources and functional abilities rather than health deficits and restrictions. The Norwegian Insurance Scheme has introduced functional ability assessments in sickness certification forms [2]. In this context, the new classification for functioning, disabilities and health (ICF) has received attention through its consistent conceptual framework for defining functional ability [3].

It is commonly found that the level of functioning tends to be poorer with increasing age and in lower social classes [4]. Eurostat surveys conducted within the European Union, have estimated the prevalence rates for moderate and severe disability in the working-age population to be 10.0% and 4.5% respectively [4]. These figures were based on self-assessed restrictions in daily activities – moderate or severe – and stricter definitions of functional limitations would give lower prevalence figures.

National and international population surveys have frequently used well-established health status instruments such as the Nottingham Health Profile [5] and the Short Form 36-item (SF-36) Health Survey [6] to assess function. Such questionnaires often have multiple aims and include several scales to measure function and quality of life. A broad array of scales might be relevant for clinical and epidemiological investigations, but are less useful in social security. To reintegrate employees in working life, there is a need for discriminating instruments that can aid the medical assessors, case managers, and labour experts in their decisions as to who should receive which types of benefits and support. Instruments based on self-report have been developed in the UK, the Netherlands, and Finland [7,8]. Self-reports of health conditions, abilities, and skills are also important approaches in the expanding research field on the relationships between health and work productivity [9]. In the WHO Health and Work Performance Questionnaire, functional status is reported, although on a more general level [10].

The Norwegian Function Assessment Scale (NFAS) is an instrument for self-report that was developed by an expert group in social insurance in 2000. It was developed to assess the need for rehabilitation, adjustment of work demands among sick-listed persons as well as the rights to social security benefits [11]. ICF was selected as a basis for facilitating multidisciplinary work and understanding, and the usage of generally accepted definition of concepts. All categories from the activities/participation component in ICF were considered, and categories not relevant for the assessment of work-related functional abilities were removed. After this process, 39 categories remained which were rephrased into questions with four response alternatives. Four response alternatives were used in preference to the five within the ICF, because fewer alternatives make the scale easier to use in assessment procedures.

The first version of the NFAS was tested for construct and convergent/divergent validity against SF-36 and the Dartmouth COOP Functional Health Assessment Charts/WONCA(COOP/WONCA), and for utility in a random sample of 386 persons sick-listed for six weeks in eight different geographical areas [11]. Based on a principal component analysis of this data, the 39 categories were regrouped into seven functional domains. Individual assessment of these domains facilitated the design of work place adjustments, and strengthened the communication between the sick-listed person and the case manager in the National Insurance Administration. Recently, the NFAS has been utilized in a study of 89 disability pensioners, to predict belief in return-to-work [12].

The final version of NFAS had good construct validity [11], but its reliability has not yet been thoroughly evaluated. The level of functional ability has yet to be assessed in the general population. This will provide important normative data necessary for score interpretation. Validity of a four- and a five-point scale version of the NFAS in a population will be reported elsewhere. The purpose of this study was to obtain normative data on the NFAS as part of The Ullensaker Study 2004, and to examine the test-retest reliability of the scale.

## Methods

### Study setting and sample

Ullensaker is a rural community which had 23,700 inhabitants in 2004. There are no major differences between the population of Ullensaker and the population of Norway with respect to demographic characteristics [13]. In 2004, postal questionnaires, which included the NFAS along with questions relating to musculoskeletal pain, were sent to all inhabitants in Ullensaker municipality in the birth cohorts 1918–20, 1928–30, 1938–40, 1948–50, 1958–60, 1968–70 and 1978–80. A randomized half of these inhabitants received the four-point version of NFAS and were included in this study. Reminders were sent at eight weeks. Information on the residential locations was given by the Population Register.

The Regional Committee for Medical Research Ethics and The Norwegian Data Inspectorate approved the study.

### Test-retest reliability

For purposes of assessing test-retest reliability, the first 30 returning a questionnaire within each of the five youngest birth cohorts were asked to complete the NFAS again at two weeks. The two oldest birth cohorts were not included because the persons are outside the normal working age in Norway of 16 to 67 years. Individuals reporting no difficulty on all NFAS items were not invited in the retest since possible changes could only be in one direction.

### The Norwegian Function Assessment Scale (NFAS)

The NFAS [11] was included in The Ullensaker Study 2004 to obtain self-reported levels of ICF based functional ability. The 39 items are relevant for assessing physical and mental functioning in working life, some relating to activities of daily living. The NFAS starts with the question "Have you had difficulty doing the following activities during the last week?" and respondents self-report 39 activities using a four-point scale from 1–4: no difficulty, some difficulty, much difficulty, could not do it. A low score indicates good functional ability.

Based on the results of principal component analysis from the previous study with sick-listed persons [11], the items comprise seven domains: Walking/standing (7 items), Holding/picking up things (8 items), Lifting/carrying (6 items), Sitting (3 items), Managing (7 items), Cooperation/communication (6 items), Senses (2 items). These domains have evidence for validity in sick-listed persons [11]. The main application of the NFAS is likely to be social insurance. Hence it was decided to keep the domains from the earlier study with sick-listed persons [11]. It should, however, be anticipated that principal component analysis based on data from the general population in Ullensaker will yield somewhat different results. Domain scores are calculated by adding the item scores and dividing by the number of items completed. The NFAS total scores are calculated by adding all 39 item scores and dividing by the number of items completed. Thus, missing values were ignored.

Demographic data about the education level was included in the questionnaire with the response categories of lower secondary school, upper secondary school (technical), upper secondary school (preparatory), university 1–4 years, university >4 years. Education level was then categorized into three groups: ≤ 9 years, 10 to 12 years and ≥13 years.

### Statistical analyses

Internal consistency was assessed by Cronbach's alpha. Test-retest reliability was assessed by calculating total proportions of agreement, weighted kappa [14], and intraclass correlation coefficient (ICC) (two-way mixed model with the measure of absolute agreement). Since data are categorical, non-parametric tests for independent samples were used to compare subgroups.

## Results

Of the 3000 questionnaires posted, 1620 (54.0%) were returned. Compared to respondents, non-respondents were more likely to be male (p < 0.001) and young or very old (Table 1). Of the respondents, 18.5%, 47.5% and 34.1% reported ≤ 9 years, 10 to 12 years and ≥13 years of education respectively.

**Table 1:** Response rates by age and gender (N = 1620)

	Number included (%)	Response rate (%)
Females	905 (55.9)	60.0
Males	715 (44.1)	48.0
		
Age:		
24–26	150 (9.3)	33.3
34–36	429 (26.5)	49.9
44–46	301 (18.6)	54.2
54–56	358 (22.1)	68.4
64–66	219 (13.5)	66.2
74–76	132 (8.1)	66.8
84–86	31 (1.9)	37.8

The mean level of missing data for the 39 NFAS items was 3.3% and 78.5% had no missing data. For the great majority of items, missing values ranged from 1.9 – 4.6%. Holding and turning a steering wheel (5.3%), driving a car (6.1%), working in groups (9.0%), and guiding others in their activities (9.3%) had higher missing values. There was a significant increase of missing values with age (p < 0.001).

Item responses were skewed towards no difficulty; range 63.5 – 96.8%. The percentage of respondents reporting no difficulty for all 39 items was 33.1%. The items going up and down stairs, engaging in your leisure activities, pushing and pulling with your arms, cleaning your house, staying alert and being able to concentrate, managing everyday stress and strains, managing to take criticism, managing to control your anger and aggression, and remembering things, represent functional activities in which more than 20% of the population reported difficulties.

Cronbach's alpha ranged from 0.67 (Sitting) to 0.91 (Walking/standing) for domains and was 0.95 for the total scores. Five of seven domains exceeded the 0.70 reliability standard for use in groups [15], the remaining two just failing to meet this criterion.

### Test-retest

Retest questionnaires were returned by 101 of the 134 (75.4%) individuals sent a second questionnaire. Most persons in the youngest cohort reported no difficulty for all questions, resulting in fewer candidates in this cohort (n = 17). The respondents were significantly older (p < 0.05) than the non-respondents, but were otherwise comparable. With the exception of four items – writing, which showed a deterioration (p < 0.01) in function, and managing to take criticism, managing to control your aggression and anger, and remembering things, which showed an improvement (p = 0.01) in function – there were no score differences between test and retest. The proportion scoring exactly the same on both occasions (total proportions of agreement) was high, ranging from 0.68 – 0.97. Weighted kappa values ranged from 0.38 (fair agreement) to 0.83 (almost perfect agreement) [16] (Table 2). The weighted kappa values for single items showed large variability, but the values for six of the seven domains were above 0.61, indicating good agreement. ICC values ranged from 0.79 (substantial) to 0.88 (almost perfect) [16] (Table 2).

**Table 2:** Weighted kappa and ICC in a test-retest study using NFAS(N = 101)

**No.**	**N**	**Weighted kappa**	**ICC^a^**
**Walking/standing**		**0.66**	**0.85**
1	99	0.47	
2	100	0.60	
3	99	0.70	
4	98	0.66	
5	100	0.65	
6	100	0.64	
7	101	0.76	
**Holding/picking up things**		**0.67**	**0.87**
8	100	0.76	
9	99	0.40	
10	100	0.83	
11	100	0.71	
12	101	0.72	
13	99	0.51	
14	99	0.57	
15	101	0.70	
**Lifting/carrying**		**0.65**	**0.82**
16	97	0.58	
17	98	0.69	
18	101	0.56	
19	99	0.64	
20	100	0.76	
21	99	0.52	
**Sitting**		**0.61**	**0.79**
22	100	0.63	
23	101	0.64	
24	99	0.58	
**Managing**		**0.57**	**0.79**
25	100	0.67	
26	96	0.75	
27	97	0.48	
28	99	0.57	
29	101	0.56	
30	97	0.51	
31	99	0.38	
**Cooperation/communication**		**0.68**	**0.88**
32	98	0.64	
33	100	0.61	
34	100	0.60	
35	101	0.74	
36	101	0.64	
37	101	0.47	
**Senses**		**0.68**	**0.80**
38	100	0.42	
39	101	0.82	

^a ^ICC = Intraclass correlation coefficient (two-way mixed with absolute agreement)

### Gender

Item and domain scores ranged from 1.04 to 1.42 and from 1.05 to 1.25 respectively (Table 3). Males reported significantly better functional ability than females on 33 items. With the exception of the Cooperation/communication domain, domain and total scores were significantly better for males than females.

**Table 3:** Descriptive statistics for NFAS items and domain scores by gender (N = 1620)

		All		Males		Females		
Domain/item^a^		(mean)	(SD)	(mean)	(SD)	(mean)	(SD)	p-value^b^
**Walking/standing:**		**1.25**	**0.47**	**1.16**	**0.37**	**1.30**	**0.51**	**<0.001**
1.	Standing	1.19	0.48	1.12	0.38	1.24	0.54	<0.001
2.	Walking less than a kilometre on flat ground	1.19	0.56	1.11	0.43	1.25	0.63	<0.001
3.	Walking more than a kilometre on flat ground	1.32	0.74	1.21	0.59	1.41	0.84	<0.001
4.	Walking on different surfaces	1.24	0.56	1.16	0.46	1.31	0.62	<0.001
5.	Going up and down stairs	1.33	0.63	1.22	0.53	1.41	0.69	<0.001
6.	Going shopping for your groceries	1.18	0.49	1.09	0.37	1.25	0.56	<0.001
7.	Putting on your shoes and socks	1.21	0.48	1.19	0.43	1.23	0.52	0.33
								
**Holding/picking up things**		**1.14**	**0.32**	**1.08**	**0.19**	**1.18**	**0.36**	**<0.001**
8.	Picking up a coin from a table with your fingers	1.10	0.37	1.07	0.30	1.13	0.41	<0.001
9.	Holding and turning a steering wheel	1.06	0.35	1.01	0.11	1.10	0.46	<0.001
10.	Driving a car	1.14	0.57	1.04	0.03	1.23	0.72	<0.001
11.	Preparing food	1.10	0.39	1.07	0.37	1.12	0.40	<0.001
12.	Writing	1.11	0.38	1.07	0.31	1.15	0.43	<0.001
13.	Performing everyday tasks on your own	1.15	0.43	1.07	0.30	1.20	0.50	<0.001
14.	Engaging in your leisure activities	1.30	0.65	1.21	0.53	1.36	0.72	<0.001
15.	Putting on and taking off your clothes	1.13	0.39	1.09	0.30	1.16	0.44	0.001
								
**Lifting/carrying**		**1.23**	**0.46**	**1.12**	**0.30**	**1.31**	**0.52**	**<0.001**
16.	Lifting an empty soda bottle crate from the floor	1.15	0.51	1.07	0.32	1.21	0.62	<0.001
17.	Carrying shopping bags in your hands	1.23	0.55	1.08	0.33	1.35	0.65	<0.001
18.	Carrying a little sack/backpack on your shoulders or back	1.20	0.56	1.09	0.36	1.29	0.67	<0.001
19.	Pushing and pulling with your arms	1.31	0.56	1.19	0.47	1.41	0.69	<0.001
20.	Cleaning your house	1.33	0.64	1.18	0.50	1.44	0.71	<0.001
21.	Washing your clothes	1.16	0.49	1.13	0.48	1.17	0.50	0.02
								
**Sitting**		**1.10**	**0.32**	**1.05**	**0.22**	**1.14**	**0.35**	**<0.001**
22.	Sitting on a kitchen chair	1.08	0.34	1.05	0.25	1.12	0.39	<0.001
23.	Riding as a passenger in a car	1.06	0.27	1.03	0.20	1.08	0.32	<0.001
24.	Riding as a passenger on public transport	1.15	0.54	1.07	0.36	1.22	0.64	<0.001
								
**Managing**		**1.25**	**0.41**	**1.19**	**0.35**	**1.29**	**0.44**	**<0.001**
25.	Staying alert and being able to concentrate	1.26	0.50	1.20	0.46	1.30	0.53	<0.001
26.	Working in groups	1.18	0.52	1.14	0.46	1.22	0.57	0.01
27.	Guiding others in their activities	1.19	0.56	1.14	0.50	1.23	0.60	0.001
28.	Managing everyday responsibility	1.15	0.41	1.10	0.35	1.19	0.46	<0.001
29.	Managing everyday stress and strains	1.33	0.58	1.23	0.50	1.40	0.46	<0.001
30.	Managing to take criticism	1.34	0.61	1.27	0.56	1.40	0.64	<0.001
31.	Managing to control your anger and aggression	1.29	0.53	1.25	0.49	1.32	0.56	0.03
								
**Cooperation/communication**		**1.18**	**0.32**	**1.16**	**0.28**	**1.18**	**0.32**	**0.25**
32.	Remembering things	1.42	0.61	1.39	0.58	1.45	0.62	0.05
33.	Understanding spoken messages	1.21	0.48	1.21	0.46	1.21	0.49	0.81
34.	Understanding written messages	1.07	0.31	1.06	0.29	1.08	0.33	0.06
35.	Speaking	1.07	0.28	1.05	0.25	1.08	0.31	0.03
36.	Participating in a conversation with many people	1.19	0.49	1.19	0.48	1.19	0.50	0.71
37.	Using the telephone	1.07	0.32	1.05	0.26	1.09	0.35	0.06
								
**Senses**		**1.05**	**0.22**	**1.03**	**0.17**	**1.06**	**0.25**	**0.02**
38.	Watching television	1.05	0.24	1.03	0.18	1.06	0.28	0.01
39.	Listening to the radio	1.04	0.26	1.03	0.20	1.05	0.30	0.20
								
**Total scores**		**1.20**	**0.31**	**1.13**	**0.21**	**1.24**	**0.34**	**<0.001**

^a ^Items use a four-point scale of no difficulty, some difficulty, much difficulty and could not do it. Domain scores and total scores are calculated by adding item responses and dividing by the number of items completed.

^b^Mann Whitney U-test.

### Age

Domain and total scores for males and females within different age groups are given in Table 4. With the exception of females in the age group 54–56, the total scores increased gradually with age (p < 0.001). With the exception of the Senses domain for males, there is a large deterioration in reported functional ability from second oldest to the oldest age group. When the oldest age cohorts of females and males were excluded, the difference in scores for the Sitting domain became insignificant. Domain scores for three of the physical domains, Walking/standing, Holding/picking up things and Lifting/carrying, had a significant gradual increase with age. Among males, the increases in scores were significant for the Cooperation/communication and Senses domains and not significant for the Sitting and the Managing domains. For females, there was no clear age gradient for the mental domains, and peaks in reporting difficulties were found in the age groups 44–46 and 84–86.

**Table 4:** Domain and total NFAS scores in males and females for different age groups (N = 1620)

	Age groups							
Domain	24–26	34–36	44–46	54–56	64–66	74–76	84–86	p-value^a^
**N of females**	90	254	158	202	108	73	19	
								
Walking/standing	1.15	1.21	1.28	1.31	1.46	1.52	2.17	<0.001
Holding/picking up things	1.08	1.13	1.20	1.20	1.22	1.31	1.88	<0.001
Lifting/carrying	1.12	1.22	1.36	1.34	1.40	1.46	2.11	<0.001
Sitting	1.09	1.11	1.15	1.17	1.12	1.13	1.70	<0.001
Managing	1.22	1.28	1.38	1.29	1.29	1.27	1.65	0.01
Cooperation/communication	1.17	1.18	1.24	1.16	1.15	1.19	1.44	0.01
Senses	1.03	1.06	1.10	1.05	1.01	1.01	1.28	0.01
								
Total scores	1.14	1.19	1.28	1.25	1.28	1.33	1.80	<0.001
								
**N of males**	62	175	143	154	109	61	12	
								
Walking/standing	1.04	1.07	1.16	1.18	1.26	1.32	1.60	<0.001
Holding/picking up things	1.04	1.04	1.06	1.10	1.12	1.12	1.27	<0.001
Lifting/carrying	1.05	1.05	1.10	1.17	1.18	1.18	1.67	<0.001
Sitting	1.03	1.03	1.04	1.09	1.05	1.02	1.08	0.60
Managing	1.15	1.18	1.18	1.23	1.20	1.14	1.52	0.16
Cooperation/communication	1.10	1.12	1.13	1.16	1.24	1.24	1.30	<0.001
Senses	1.01	1.01	1.02	1.04	1.04	1.08	1.08	0.003
								
Total scores	1.07	1.08	1.12	1.16	1.18	1.18	1.42	<0.001

^a^Kruskal-Wallis test with age group as grouping variable

### Education

NFAS scores decreased with more years of education, indicating better self-reported functional ability (Table 5). With the exception of the Senses domain these differences were significant. When splitting the data in males and females and age cohorts, the association between education and Sitting domain disappeared (p > 0.05). For the other three physical domains and for the Managing domain, the gradient for education remained evident (p < 0.05) in age cohorts 44–76 among males and 24–56 among females. For the Cooperation/communication domain the association with functional ability was significant among females aged 24–46, but not among males.

**Table 5:** Domain and total NFAS scores in participants with different education levels (N = 1620)

	Years of education			
Domain	≤9	10 to 12	≥13	p-value^a^
N^b^	299	776	524	
				
Walking/standing	1.42	1.25	1.14	<0.001
Holding/picking up things	1.24	1.14	1.08	<0.001
Lifting/carrying	1.36	1.25	1.13	<0.001
Sitting	1.14	1.11	1.07	<0.001
Managing	1.38	1.26	1.17	<0.001
Cooperation/communication	1.25	1.19	1.11	<0.001
Senses	1.06	1.05	1.03	0.06
				
Total scores	1.31	1.20	1.12	<0.001

^a ^Kruskal-Wallis test with years of education as grouping variable

^b ^Missing data about education for 21 persons

## Discussion

The Norwegian Function Assessment Scale (NFAS) was developed by an expert group to ensure that the instrument has content validity, as a measure of functional ability relevant to the working population. With just 39 items the NFAS is suitable for inclusion in population surveys with minimum respondent burden and take an estimated ten minutes to complete. The instrument seems to be acceptable to the general population in Norway, even though the response rate was relatively modest in some age cohorts. The response rate represents a potential study limitation as we do not know the possible effect imposed by the non-respondents. Compared with national population data [13], the study sample included fewer persons in the youngest and the oldest cohort. Since these two groups are at the opposite ends of the functional ability continuum, the effects on scores might to some extent be cancelled out. Further, more females than males returned the questionnaire, which might have led to poorer scores than if all responded. On the other hand, this effect may have been lessened by the higher percentage of persons with education at university level in the sample compared to the distribution of educational level in the whole population [13].

Levels of missing data were within acceptable limits. However, a few items had a high percentage of missing values, which is probably because there was no "not applicable" option. When a participant considered a functional activity irrelevant, he or she would probably have left this item unanswered. Some items could have been irrelevant for the two oldest cohorts since many of these participants have retired from work or do not drive a car. Including a not applicable option might have lowered missing values for some items.

The NFAS was originally developed for persons of working age. The small number of participants in the two oldest age cohorts, and the poorer data quality among these respondents due to more missing values and irrelevant items imply that caution should be exercised when using these normative data on groups outside the working age. Otherwise, the data quality was acceptable.

### Reliability

The level of Cronbach's alpha was acceptable with two of the domains only just failing to meet the criterion of 0.70 for use in groups of people [15]. The participants received the test and the retest questionnaires about two weeks apart. In this way the recall bias might be minimal, but there may have been a real change in health related function. Functional health status is also likely to show some day-to-day variation. For the most part, mean changes were fairly evenly distributed between improvements and deteriorations.

The total proportions of agreement in this test-retest was high compared to a study examining test-retest reliability of COOP/WONCA [17]. Compared to a further test-retest study using the COOP/WONCA charts [18], the weighted kappa values were slightly lower. The ICC values for domains indicated substantial to almost perfect agreement, and all met the reliability standard of 0.70 for use in groups [15]. Compared with other studies using the SF-36 [19,20], ICC values were similar. Overall the test-retest reliability is acceptable.

### Normative data

As expected, the data were highly skewed indicating that a large proportion of the population did not experience difficulties with functional activities. One in three respondents reported no difficulty on all items indicating excellent functional ability, and the remaining two thirds reported a variety from minor to major difficulties with different functional activities. The population seems to have most problems with remembering and least problems with their senses. Walking/standing and Managing domain have the highest scores, whereas Senses and Sitting the lowest. The items, watching television and listening to the radio, had very low scores, indicating that very few respondents reported difficulties with this. However, problems with these senses are important aspects in relation to work.

Men reported higher functional ability than women on most items. The findings of previous studies differ somewhat, which may, at least partly, be due to the use of different instruments and the aspects of health that they measure. Of the studies looking at functional health status using the SF-36, five had a similar conclusion [21-26], whereas one study did not [27]. According to one study using the COOP/WONCA charts, males reported better functional ability than females on the first four of the six charts [28]. The report by Grammenos [4], did not show systematically significant differences between the percentages of men and women of working age in the European Union reporting disability.

The significant age gradient in physical domains and the non-gradient in mental domains found in this study follows previous research [21,23,24,26-28]. Grammenos [4] also found a strong non-linear age gradient in the reported disability prevalence rates in the European Union. In our study, females aged 44–46 reported more difficulties on mental domains than younger or older females, the exception being the oldest cohort. For males, a peak at the age group 54–56 was found for the Managing domain only. These findings are supported by the results from a study by Hensing et al [29] showing that the cumulative incidence of sickness absence for a psychiatric diagnosis was highest among those aged 45–59. The association between age and functional ability seems to be more complex in mental domains than in physical domains.

In this study, the length of education was significantly related to functional ability level with better levels among the persons with the highest levels of education. This finding is supported by previous studies [22-25]. In the European Union report [4], education was inversely associated with disability in all countries. Further, positive correlations between income and health, and a presence of collinearity between education, income and socio-economic status were reported. After adjusting for gender and age, we only found associations between educational level and reported functional ability for some subgroups in our study, whereas Sullivan et al [23] reported significant gradients after adjusting for age. The relations between age, gender, education, and income are often difficult to disentangle. In older generations of women, their well-being is more likely to be influenced by their husbands' education and income. The lack of association between functional ability and education in younger men is likely explained by young men's general high functional levels. We propose that a normative population data set must take age, gender, and education into account.

### Comparisons with sick-listed persons

Comparing this population study data with data from the sample with 386 Norwegians sick-listed for six weeks [11], the population sample scores are lower than for the sick-listed persons. The largest difference for domains is for the Lifting/carrying domain (1.23 vs. 1.85), and for Walking/standing, Holding/picking up things and Managing there are 0.31 to 0.35 differences in domain scores for the two samples. Senses had the lowest mean value in both samples (1.05 vs. 1.13). The total score for the sick-listed persons was 1.52 as compared to 1.20 for this population study sample. Looking at single items, the four with the highest difference between the two samples, were engaging in your leisure activities (1.39 vs. 2.28), cleaning your house (1.33 vs. 2.23), carrying shopping bags in your hands (1.23 vs. 1.96) and managing everyday stress and strains (1.33 vs. 1.99). These four functional activities seem to imply much more difficulties for the sample of sick listed than for the normal population. In the sample of 386 sick-listed persons [11] no significant differences between males and females nor any age gradient were found, as opposed to the normal population where females and older persons report more difficulties with functional activities than males and younger persons.

## Conclusion

This study presents population scores on the NFAS by gender, age and length of education. Data quality, internal consistency and test-retest reliability were acceptable. The main findings were that females, older persons and persons with lower levels of education reported more functional problems than males, younger persons and persons with higher levels of education. A large proportion of the respondents reported no difficulty for most items and very few answered that they could not do it. The domains, in which the respondents reported most problems with functional activities, were Walking/standing, Lifting/carrying and Managing. These data will serve as basis for the development of national population norms.

## Competing interests

The author(s) declare that they have no competing interests.

## Authors' contributions

NØ planned and designed the study, performed some of the statistical analysis, drafted the manuscript and coordinated the study. SB planned and designed the study, participated in the interpretation of results and in drafting and revising the manuscript. AG helped in the interpretation of the results and participated in drafting the manuscript. JSB performed most statistical analysis and reviewed the manuscript. BN participated in planning and designing the study, collected the data and participated in drafting the manuscript. PG participated in the planning and design of the study, interpretation of the results and in drafting the manuscript. All authors read and approved the final manuscript.

## Pre-publication history

The pre-publication history for this paper can be accessed here:


